# Incidence Trends of Cervical Cancer and Its Precancerous Lesions in Women of Central Switzerland from 2000 until 2014

**DOI:** 10.3389/fmed.2018.00058

**Published:** 2018-03-16

**Authors:** Katrin Ochs, Gesine Meili, Joachim Diebold, Volker Arndt, Andreas Günthert

**Affiliations:** ^1^Department of Obstetrics and Gynecology, Cantonal Hospital of Lucerne, Lucerne, Switzerland; ^2^Institute of Pathology, Cantonal Hospital of Lucerne, Lucerne, Switzerland; ^3^Foundation National Institute for Cancer Epidemiology and Registration (NICER), Zurich, Switzerland

**Keywords:** cervical cancer, cervical intraepithelial neoplasia, conizations, Switzerland, incidence, screening

## Abstract

**Objective:**

Cervical cancer (CC) screening by Pap smears has led to a decrease in the incidence of CC worldwide. Indeed, the incidence of CC in Switzerland is very low; however, there is a lack of data to evaluate the efficiency of the Pap smear as a screening tool. Until now, only Pap smears have been used and other methods such as the presence of an infection with HPV have not been integrated into the routine screening. The aim of this study is to evaluate trends in the incidence of CC and its precancerous lesions in Central Switzerland, which represents a rural region, with those in urban regions and the entire country of Switzerland.

**Methods:**

All conizations and CC registered between 2000 and 2014 at the Institute of Pathology at the Cantonal Hospital of Lucerne have been included in our study. The incidence of CC and its precancerous lesions have been categorized according to age, stage, morphology, and study period. Age-standardized incidence in the Canton of Zurich and the entire country served as reference for the assessment of trends in CC incidence in the study region.

**Results:**

In Central Switzerland, the number of conizations performed annually has more than doubled over the observed 15 years. There has been a significant increase in precancerous lesions, which were found in approximately 50% of conizations. The total number of CC diagnosed by conization increased by 37.5% and the total of CIN3 increased by 130%. Age-standardized incidence of CC and CIN3 increased from 2.4 to 3.3/100,000 and from 11.6 to 26.9/100,000, respectively. The incidence of CC was lower in Central Switzerland compared to incidence in the Canton of Zurich and in Switzerland generally.

**Conclusion:**

Approximately 50% of all conizations were performed on women without serious precancerous lesions. For this reason, we recommend the adaptation of screening modalities and the use of risk stratification to avoid overtreatment. In light of the forthcoming implementation of an HPV vaccination program, our data provides important baseline information.

## Introduction

Since the inception of cervical cancer (CC) screening by regular Pap smears, the incidence of CC has decreased significantly worldwide. Since the 1980s, the incidence of CC was reduced by almost 50% in Switzerland. Nowadays, there is an incidence of 3.6/100,000 in Switzerland, which constitutes a very low rate within the European context (e.g., Germany had an incidence of 8.2/100,000 in 2012) (1). The current guidelines for CC screening in Switzerland recommend that for women with abnormal cytology in the Pap smear, the performance of a colposcopy, and if the finding looks abnormal a biopsy. Women with a pathological cytology indicating high-grade squamous intraepithelial lesion or pathology [high-grade cervical intraepithelial lesion (CIN) or adenocarcinoma (AC) *in situ*] are treated by conization (2). There is no valid data available on the amount of conizations performed in Switzerland, but it is estimated at 3,000 per year (3). The proportion of different findings in conizations is unknown and there is also a lack of information on CC occurrence in rural and urban regions of Switzerland.

The main aim of this study is to assess the incidence of CC and its precancerous lesions in Central Switzerland. We evaluated changes regarding histological subtypes of CC, proportion of CC diagnosed through conization as well as mean age at time of diagnosis of CC or performance of conization. As Central Switzerland is a rural area, trends in CC incidence in the study region were compared with data for all of Switzerland and for the Canton of Zurich, to compare rural and urban parts of Switzerland.

## Materials and Methods

### Data Sources

Data were provided by the Institute of Pathology at the Cantonal Hospital of Lucerne, which receives its samplings from the Cantons of Central Switzerland including Lucerne, Obwalden, Nidwalden, Uri, Schwyz, and Zug. In 2014, Central Switzerland had 782.374 residents, representing 9.5% of the Swiss population.

All 174 cases of CC registered at the Institute of Pathology at the Cantonal Hospital of Lucerne between 2000 and 2014 were analyzed. Data included year and age at time of diagnosis, histopathological type, nationality, and FIGO stage (4). FIGO stage was divided into four categories: FIGO IA, IB, II, and >II. In three cases, data on FIGO stage were not available. Histopathological type was classified into three categories: squamous cell carcinoma (SCC), AC, and other types. We classified adenosquamous carcinoma, adenoid cystic carcinoma, neuroendocrine carcinoma, and cervical carcinosarcoma as other types.

Furthermore, we analyzed all conizations registered between 2000 and 2014 at the Institute of Pathology at the Cantonal Hospital of Lucerne (*n* = 2,005). Data included findings as well as year and age at time of conization. Results were classified as negative for dysplasia or malignancy, CIN1, CIN2, CIN3, and CC.

To evaluate whether our institution-based data represented the population-based incidence rate of Central Switzerland, we compared data of the Swiss national dataset managed by the National Institute for Cancer Epidemiology and Registration (NICER) to data of the Institute of Pathology at the Cantonal Hospital of Lucerne.

The comparison had to be restricted to the years between 2010 and 2012 since the population-based cancer registry for Central Switzerland has only been established very recently. Although the region of Central Switzerland usually includes the Cantons of Lucerne, Nidwalden, Obwalden, Uri, Schwyz, and Zug, the population-based cancer registry of Central Switzerland comprises only the Cantons of Lucerne, Nidwalden, Obwalden, and Uri but not Schwyz and Zug. Both numerators and denominators for the calculation of population-based incidence rates have been adjusted accordingly and pertain to Central Switzerland excluding Schwyz and Zug. To find out if there are similar trends in CC incidence, we compared age-standardized incidence rates (ASIR) of CC in Central Switzerland (excluding Schwyz and Zug) with the corresponding ASIR in Switzerland, extracted from the Swiss national dataset managed by NICER. A very recent and extensive evaluation demonstrated high completeness across all registries and for most cancer types including CC (5). In addition, to analyze if there is a difference in ASIR of CC between rural and urban regions of Switzerland, we compared population-based data of Central Switzerland (excluding Schwyz and Zug) with data of the Canton of Zurich, also provided by NICER. According to the list of “areas with urban character 2012” compiled by the Swiss Federal Statistical Office, the Canton of Zurich belongs to an area with urban characteristics whereas most parts of Central Switzerland belong to the rural area of Switzerland (6). Data for Switzerland and the Canton of Zurich were only available for the time period between 2000 and 2013.

Cases from the Cantons of Zug and Schwyz were excluded from analysis due to potential underrepresentation in the database of the Cantonal Hospital of Lucerne. Presumably, underrepresentation is due to the fact that women from these two cantons went to Zurich for treatment since Zug and Schwyz are very close to Zurich. Furthermore, according to the FSO, the Cantons of Zug and Schwyz do not constitute rural areas.

Ethical approval is not required because of the retrospective character of the study. We used anonymized datasets only.

### Statistical Analysis

Patients were classified into the following age groups: <29, 30–39, 40–49, 50–59, 60–70, and >70 years. ASIR for CC and CIN3 were calculated for every year in the period between 2000 and 2014 using the European standard population. For the descriptive analysis, cases were stratified into 5-year time periods (2000–2004, 2005–2009, and 2010–2014). Poisson regression was used to test for linear trend in 5-year incidence of CC and CIN3. The data analysis was carried out using SPSS statistical software (Statistical Package for Social Sciences, version 22.0, SPSS Inc., Chicago, IL, USA). The Kruskal–Wallis test was conducted to test for significance of mean age and FIGO stage as well as mean age and degree of dysplasia in conizations. The significance level was set at 5%.

## Results

### Trends in Findings in Conizations in Central Switzerland

**Figure 1:**
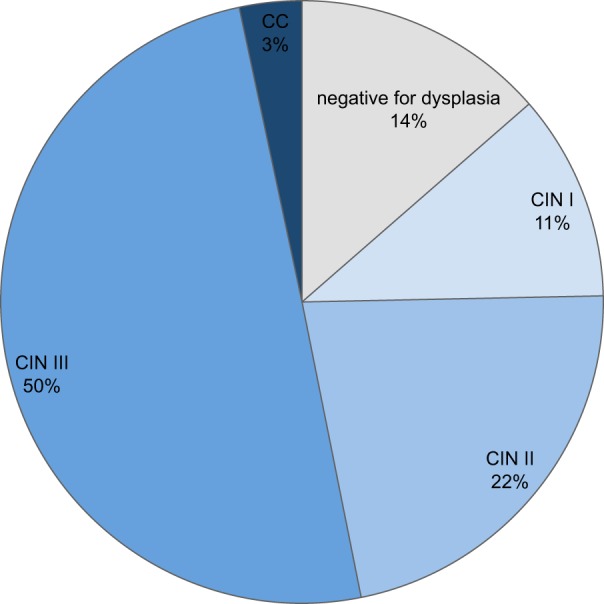
Findings in conizations in Central Switzerland, 2000–2014. In total, 272 conizations showed no evidence of dysplasia, 220 showed CIN1, 443 CIN2, 1,000 CIN3, and 70 showed cervical cancer (CC).

**Figure 2:**
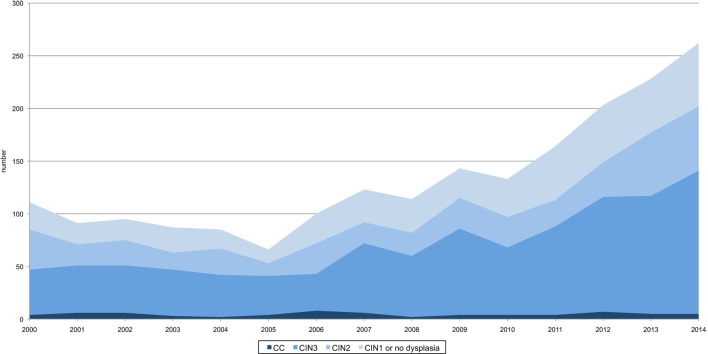
Number and proportions of dysplastic findings and cervical cancer (CC) diagnosed by conization in Central Switzerland, 2000–2014. There is a significant increase of conizations (*n* = 2,005) as well as CIN3 and CIN2 diagnosed by conization (*p*-value < 0.001, respectively). CC shows a stable course.

Between 2000 and 2014, a total of 2005 conizations were performed in Central Switzerland. Overall, 272 conizations (13.6%) showed no evidence of dysplasia, 220 (11.0%) were classified as CIN1, 443 (22.1%) as CIN2, 1,000 (49.9%) as CIN3, and 70 (3.5%) as CC (Figure 1). The annual number of conizations (range 66–262) sharply increased during the study period (*p*-value < 0.001). Also, the absolute number of CIN3, CIN2, CIN1, and findings without dysplasia increased proportionally. Only the proportion of invasive CC decreased from around 5% at the beginning to around 2% at the end of the study period (Figure 2).

The mean age of women at time of conizations was 36.0 years (range 18–81 years), but varied according to degree of dysplasia (*p*-value < 0.001). Invasive CC and conizations with no evidence of dysplasia were found more often in older women (median 41.1 and 40.3 years). Mean age at time of diagnosis of CIN1, CIN2, and CIN3 was comparable with 35.5 vs. 34.7 vs. 35.2 years.

### Incidence Trends of Invasive CC and Precancerous Lesions in Conizations in Central Switzerland

**Figure 3:**
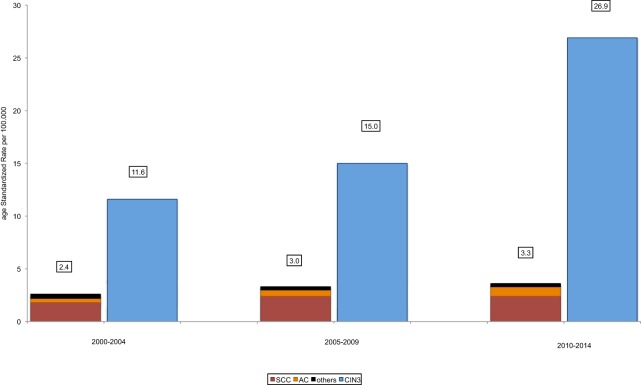
Trends in age-standardized incidence of cervical cancer (CC) and CIN3 in conizations in Central Switzerland, 2000–2014. There is a trend toward increasing age-standardized incidence rates (ASIR) of CC (*p*-value 0.075) and a significant increase of ASIR of CIN3 (*p*-value < 0.001). There is no significant increase of ASIR of squamous cell carcinoma (SCC) from 1.8/100,000 between 2000 and 2004 to 2.4/100,000 between 2005 and 2009 and between 2010 and 2014 (*p*-value 0.132) and of adenocarcinoma (AC) from 0.4/100,000 between 2000 and 2004 to 0.6/100,000 between 2005 and 2009 to 0.9/100,000 between 2010 and 2014 (*p*-value 0.423).

During the period between 2000 and 2014, 174 cases of CC were registered for Central Switzerland, corresponding to an ASIR of 2.9/100,000 females. CC incidence increased by 37.5% from 2.4/100,000 between 2000 and 2004 to 3.3/100,000 between 2010 and 2014 (*p*-value 0.075). Out of 174 CC, 134 cases were histopathologically classified as SCC (77.0%), 32 as AC (18.4%), and 8 as other subtypes of CC (4.6%). No significant change occurred in any of the subtypes over the course of the observed 15 years. In the same time, a total of 1,000 cases with CIN3 were diagnosed (ASIR 17.8/100,000). The incidence of CIN3 increased from 11.6/100,000 between 2000 and 2004 to 26.9/100,000 between 2010 and 2014 (+130%, *p*-value < 0.001) (Figure 3).

### Trends in CC Diagnosed by Conization in Central Switzerland

Cervical cancer was diagnosed by conization in 70 cases (40.2% of CC). The proportion of CC diagnosed by conization decreased from 46.7% between 2000 and 2004 to 40.0% between 2005 and 2009 to 36.2% between 2010 and 2014 (*p*-value 0.828). If CC was diagnosed by conizations, histological findings more often showed SCC (85.7 vs. 71.1%) and more rarely AC (11.4 vs. 23.1%) (OR = 2.43, *p* = 0.04). Irrespective of histological subtype, patients whose CC was diagnosed in the context of a conization were approximately 12 years younger than other CC patients (Table 1).

**Table 1:** Number and mean age of histological subtypes of cervical cancer (CC) depending on type of diagnosis, CC diagnosed by conization and CC diagnosed other than by conization.

	CC		CC n.s.		CC in conization	
	Number (%)	Mean (years)	Number (%)	Mean (years)	Number (%)	Mean (years)
Total	174	48.5	104	53.4	70	42.1
Squamous cell carcinoma	134 (77.0)	47.7	74 (71.1)	52.8	60 (85.7)	41.4
Adenocarcinoma	32 (18.4)	49.1	24 (23.1)	52.9	8 (11.4)	37.9
Others	8 (4.6)	59.3	6 (5.8)	62.7	2 (2.9)	49.0

There is no significant difference in age at time of diagnosis between the histological subtypes in all observed CC (*p*-value 0.078) or CC diagnosed by conization (*p*-value 0.279). (ns: diagnosis not elsewhere specified, diagnosis not performed by conization.)

### Trends in CC Depending on FIGO Stage in Central Switzerland

**Figure 4:**
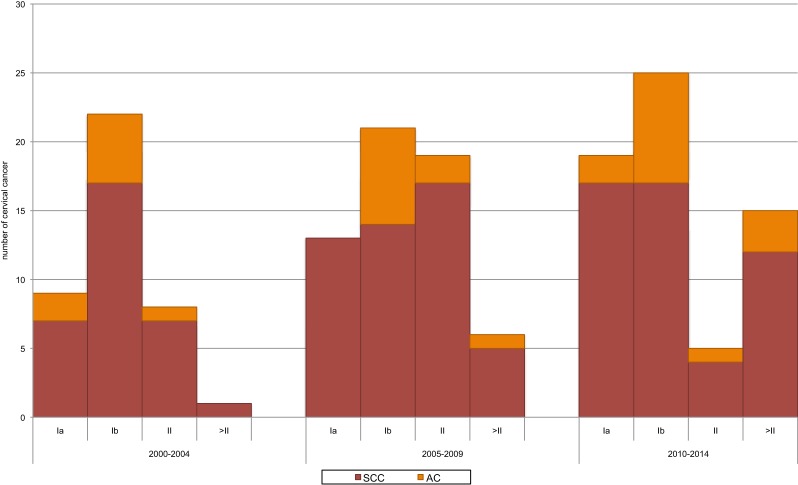
Trends in number of histological subtypes of cervical cancer (CC) by FIGO stage at time of diagnosis, Central Switzerland 2000–2014. There is a trend of increasing numbers of findings with FIGO IA (*p*-value 0.077) and a significant increase of findings FIGO >II (*p*-value < 0.001). If CC was diagnosed in FIGO IA, adenocarcinoma (AC) was found in 9.8% and squamous cell carcinoma (SCC) in 90.0%, compared to FIGO IB with 29% of AC and 71% of SCC.

Overall, CC was diagnosed in FIGO IB in 40.8% of all CC cases. FIGO IA was found in 24.7%, FIGO II in 19.5%, and FIGO >II in 13.2%. The proportion of cases diagnosed with FIGO >II increased from 2 to 23% in the course of the 15-year study period (*p*-value < 0.001) (Figure 4). If diagnosis was performed by conization, the most frequent FIGO stage was FIGO IA (57.1%), followed by FIGO IB (40.0%) compared to diagnosis other than conization, which was diagnosed the most frequent in FIGO IB (41.3%) followed by FIGO II (31.7%). CC in FIGO IA was diagnosed by conization in 93.0% of cases. Women diagnosed with CC in FIGO IA were youngest, with 42.0 years, followed by FIGO IB with 45.6 years, FIGO II with 55.5 years, and FIGO >II with 60.4 years (*p*-value < 0.001).

### Comparison of CC Incidence between Central Switzerland and Switzerland and between Rural and Urban Parts of Switzerland

**Figure 5:**
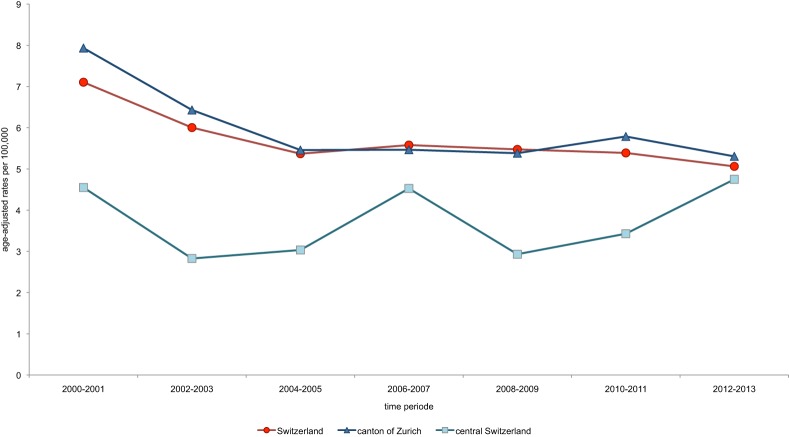
Trends in age-standardized incidence rates (ASIR) of cervical cancer (CC) in Switzerland, the Canton of Zurich and Central Switzerland. There are similar trends in ASIR of CC in Switzerland and in the Canton of Zurich over the course of the observed time. CC incidence in Central Switzerland was lower in earlier years, but all rates converged in 2012/2013 (Central Switzerland: 4.8/100,000, Switzerland: 5.1/100,000, and Canton of Zurich: 5.3/100,000).

Incidence of CC in the period between 2000 and 2013 was lower in Central Switzerland (ASIR 3.7/100,000 females) than in Switzerland overall (ASIR 5.8/100,000 females) or in the Canton of Zurich (ASIR 6.0/100,000 females). Over the course of the observed time period, CC incidence declined in all of Switzerland as well as in the Canton of Zurich. Trends and the extent of CC incidence in these two regions were almost identical. By contrast, incidence of CC in Central Switzerland was generally lower. There was, however, a tendency to increase over the years. In 2012/2013, the incidence rates of the three study regions converged (Figure 5). CC incidence rates between 2010 and 2012 in Central Switzerland, provided by NICER, and rates based on cases reported to the Institute of Pathology at the Cantonal Hospital Lucerne were identical (ASIR 3.8/100,000), indicating comparable population-based coverage for the study region.

## Discussion

Our data shows an extremely low incidence rate of CC compared to worldwide standards (1). In contrast to global trends, we found an upward trend of CC occurrence over the course of the observed 15 years (7–9). Compared to Switzerland as a whole and to the Canton of Zurich, which represents an urban part of Switzerland, the incidence rates of CC were low in Central Switzerland. This low incidence might be related to a smaller percentage of immigrants in Central Switzerland (median of 16.1%), as compared to the Canton of Zurich and to Switzerland (22.9 and 21.1%, respectively) (10). Likewise, differences in spread of risk factors for CC (e.g., contamination with HPV) or levels of participation in screening programs in relation to education and income levels might be discussed, but pertinent data are lacking. Data regarding the spread of HPV in Switzerland have been recently described in the CIN3+-study, but only six cantons had participated in it (11). The study shows that 61.8% of cervical biopsies were positive for HPV 16 and 18, and 89.5% of biopsies showed positive results for HPV-types, which are included in the nonavalent vaccine (HPV 16, 18, 31, 33, 45, 52, and 58). Unfortunately, this vaccine is not available in Switzerland yet. There are currently two HPV vaccines available. A bivalent vaccination targets HPV 16 and 18 and a quadrivalent vaccination targets HPV 6, 11, 16, and 18. Data obtained from large cohorts of women several years after implementation of bivalent (12) or quadrivalent (13) vaccination showed high efficacy against infections due to HPV type 16 and 18 as well as anogenital warts. It is obvious that HPV vaccination affects CC screening and screening modalities must be reconsidered in terms of adding HPV testing to cytology as primary screening or replace it. Until now, an HPV-based screening is not approved by the government in Switzerland.

A limitation of our analysis is the relatively small sample size of women diagnosed with CC and consequently low power. In addition, our data only shows the number of precancerous lesions diagnosed by conization while the absolute number of precancerous lesions in Central Switzerland is not analyzed. We assume this number to be very small. It is reasonable to assume that these women underwent off-label-use therapies based on imiquimod or similar treatments. The selection bias, too, may be deemed detrimental to the significance of the study. In interpreting the data from registry-based and institution-based datasets, the limitations of retrospective analyses must be considered in terms of cleanliness and completeness. However, we did succeed in proving that the institution-based data for Central Switzerland provided by the Institute of Pathology at the Cantonal Hospital of Lucerne is similar to the estimates derived from the official Swiss national dataset managed by NICER.

Our analysis shows a vast increase of conizations over the observed 15 years. The number of conizations more than doubled. However, the female population shows only a small increase of 13% (10). In total, we diagnosed a significant increase of precancerous lesions as well as of findings without dysplasia. But if we look at the amount of findings relative to the increased number of conizations, we find a constant proportion of CIN3, CIN2, CIN1, and samplings without dysplasia. It is only the proportion of CC diagnosed by conizations, which is decreasing. Consequently, the increased number of conizations seems to be justified. Considering the increasing number of samplings with CIN3 as an expression of improvement in quality in CC screening, the amount of CIN1 and CIN2 should decrease as well. This means that there is an overtreatment regarding the samplings with CIN1 and CIN2. The question arises whether the overtreatment is a result of incorrect material recovery, workup or assessment in Pap smears, biopsy or cone biopsy, or instead attributable to ignored guidelines.

It is reported that Pap smear screening seems to be less effective for AC detection than for SCC (7, 14, 15). Our results confirm this assertion. Comparing the different histological subtypes, less AC is diagnosed by conizations and, in addition, AC is frequently diagnosed at more advanced stages. The current guidelines of the Swiss Society of Obstetrics and Gynecology recommend age-appropriated screening programs through Pap smears. The use of HPV testing in screening strategies is not yet routinely recommended. HPV testing is only recommended to be used in cases of atypical squamous cell of undetermined significance and atypical glandular cells of undetermined significance (2). However, the guidelines of the American Society for Colposcopy and Cervical Pathology from 2012 recommend co-testing with HPV testing and cytology every 5 years between 30 and 64 years (16). HPV testing is known to be more sensitive, but less specific than cytology for the detection of cervical precancerous lesions (17). Recent studies show that with the addition of HPV testing to cytology, high-grade lesions are detected earlier and the incidence of grade 2 or 3 intraepithelial neoplasia or cancer can be reduced (18). This results in longer screening intervals with similar or even lower incidence cancer rates compared to cytology alone at shorter screen intervals (19–21). Recent studies have also reported that the addition of HPV testing to cytology increases the detection of women with AC and its precursors (22, 23).

Less than half of CC was diagnosed by conization and the number of CC diagnosed in an advanced stage of cancer has been increasing. The question is, however, what are the reasons behind the large number of CC not diagnosed by conizations. It may either be due to failures in the diagnostic chain or the result of many women not participating in cervical screening programs. Our data supports the assumption that women at risk do not participate in the screening programs and are then only diagnosed in advanced stages of CC. Studies have shown that organized CC screening achieves high quality standards and greater participation among the target population (24, 25). However, the implementation of an organized screening program might hardly be possible in a federal country like Switzerland.

In conclusion, our data shows that a high number of conizations are performed unnecessarily, particularly among young women. To improve CC screening, the main challenge lies in both modifying screening facilities and getting women at risk to participate in screening programs. The current guidelines for age-appropriate cervical screening programs must be revised and the implementation of routinely performed HPV tests ahead of conizations—particularly in the case of young women—should been taken into consideration.

## Author Contributions

AG conceived of the presented idea and supervised the project. KO analyzed the data and performed the calculations. VA contributed to the analysis and interpretation of the data and improved the content of the work. KO wrote the manuscript with support from GM, VA, and AG. All authors contributed to the interpretation of the results.

## Conflict of Interest Statement

The authors declare that the research was conducted in the absence of any commercial or financial relationship that could be construed as a potential conflict of interest.
